# Supplementary material for: Quantum equilibrium propagation for efficient training of quantum systems based on Onsager reciprocity
Source: Nat Commun. 2025 Jul 17;16:6595. doi: 10.1038/s41467-025-61665-6 (PMC12271321; doi:10.1038/s41467-025-61665-6)
Supplement: Supplementary file 1 — Supplementary Information [file 41467_2025_61665_MOESM1_ESM.pdf]

Supplementary Material for: Quantum Equilibrium Propagation for efficient training of quantum systems based on Onsager reciprocity

Clara C. Wanjura
Max Planck Institute for the Science of Light, Staudtstraße 2, 91058 Erlangen, Germany

Florian Marquardt
*Max Planck Institute for the Science of Light, Staudtstraße 2, 91058 Erlangen, Germany and
Department of Physics, University of Erlangen-Nuremberg, 91058 Erlangen, Germany*
(Dated: June 21, 2025)

I. DERIVATION OF ONSAGER RECIPROCITY AND THE GRADIENT FORMULA

Onsager reciprocity generally holds in equilibrium quantum and classical systems. Here, we recall how its version for the static linear response can be derived for non-degenerate pure eigenstates of closed quantum systems. First, we take the derivative, w.r.t. λ_j, of the expectation value $\langle \hat{A}_\ell \rangle \equiv \langle \Psi_n(\lambda) | \hat{A}_\ell | \Psi_n(\lambda) \rangle$ computed from the n-th eigenstate $|\Psi_n(\lambda)\rangle$ of the Hamiltonian

$$\partial_{\lambda_j} \langle \hat{A}_\ell \rangle = (\partial_{\lambda_j} \langle \Psi_n(\lambda)|) \hat{A}_\ell |\Psi_n(\lambda)\rangle + \langle \Psi_n(\lambda)| \hat{A}_\ell \partial_{\lambda_j} |\Psi_n(\lambda)\rangle = 2\mathrm{Re}\left\{ (\partial_{\lambda_j} \langle \Psi_n(\lambda)|) \hat{A}_\ell |\Psi_n(\lambda)\rangle \right\} \tag{1}$$

in which we used that $\hat{A}_j = \hat{A}_j^\dagger$. The above expression involves derivatives of the eigenstate $|\Psi_n(\lambda)\rangle$. Projecting the derivative into the eigenbasis, we obtain

$$\begin{aligned} \partial_{\lambda_j} |\Psi_n(\lambda)\rangle &= \sum_m |\Psi_m(\lambda)\rangle\langle\Psi_m(\lambda)| \partial_{\lambda_j} |\Psi_n(\lambda)\rangle \\ &= \sum_{m \neq n} \frac{|\Psi_m(\lambda)\rangle\langle\Psi_m(\lambda)| \hat{A}_j |\Psi_n(\lambda)\rangle}{E_n(\lambda) - E_m(\lambda)} + |\Psi_n(\lambda)\rangle\langle\Psi_n(\lambda)| \partial_{\lambda_j} |\Psi_n(\lambda)\rangle \,. \end{aligned} \tag{2}$$

The first term on the right-hand side stems from first order perturbation theory, the second term accounts for gauge freedom, i.e., $|\Psi_n(\lambda)\rangle$ could depend on an arbitrary phase which itself depends on λ. Note that the last term is fully imaginary since

$$0 = \partial_{\lambda_j} \langle \Psi_n(\lambda) | \Psi_n(\lambda) \rangle = \left\langle \partial_{\lambda_j} \Psi_n(\lambda) \middle| \Psi_n(\lambda) \right\rangle + \left\langle \Psi_n(\lambda) \middle| \partial_{\lambda_j} \Psi_n(\lambda) \right\rangle \tag{3}$$

which implies $\left\langle \partial_{\lambda_j} \Psi_n(\lambda) \middle| \Psi_n(\lambda) \right\rangle = -[\left\langle \partial_{\lambda_j} \Psi_n(\lambda) \middle| \Psi_n(\lambda) \right\rangle]^*$, so $\mathrm{Re} \left\langle \partial_{\lambda_j} \Psi_n(\lambda) \middle| \Psi_n(\lambda) \right\rangle = 0$. Inserting the above expression into Eq. (1), we find

$$\partial_{\lambda_j} \langle \hat{A}_\ell \rangle = 2\mathrm{Re}\left\{ \sum_{m \neq n} \frac{\langle \Psi_n(\lambda)| \hat{A}_j |\Psi_m(\lambda)\rangle \langle \Psi_m(\lambda)| \hat{A}_\ell |\Psi_n(\lambda)\rangle}{E_n(\lambda) - E_m(\lambda)} \right\} . \tag{4}$$

Since this expression is symmetric under changing j and ℓ, this results in Onsager reciprocity for the static linear response

$$\partial_{\lambda_j} \langle \hat{A}_\ell \rangle = \partial_{\lambda_\ell} \langle \hat{A}_j \rangle . \tag{5}$$

II. PHASE SENSING: ANALYSIS OF SENSOR-SYSTEM COUPLING EFFECTS

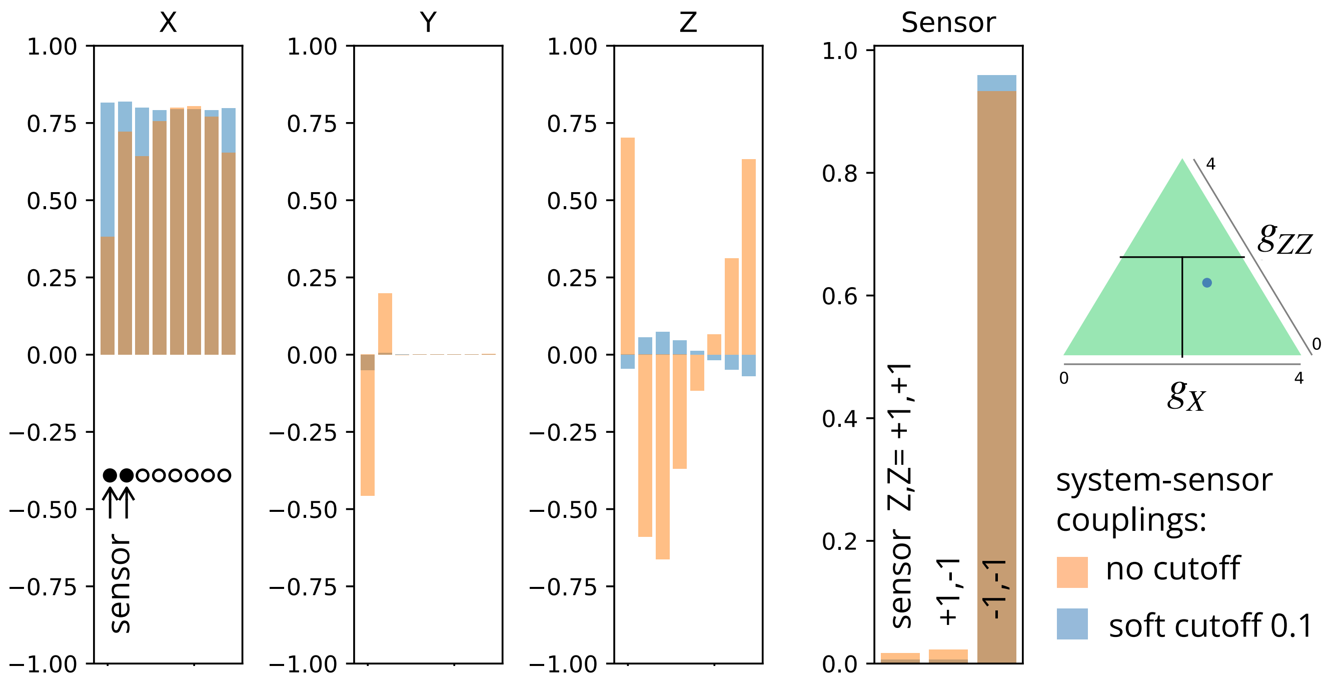

Supplementary Fig. S1. Phase recognition: Influence of the sensor on the quantum many-body system. We show the spatial profile of the expectation values of spin operators in the cluster Ising model chain of length 8 ($\langle \hat{X}_j \rangle$, $\langle \hat{Y}_j \rangle$, and $\langle \hat{Z}_j \rangle$). In the absence of coupling to the phase sensor quantum system, all profiles would be translationally invariant, i.e. constant (zero for Y and Z in this case). The sensor, whose location along the chain is indicated in the first panel, leads to deviations in its vicinity. These deviations depend on the strength of the sensor-system coupling, which can be suppressed using a cutoff during training. Panel marked 'sensor': probabilities of measuring the two qubits of the sensor in one of the three configurations that are trained to indicate the phase. Both with and without cutoff, the correct phase is indicated. Triangle on the right: for the present figure, the parameters of the Ising model were chosen in the vicinity of a phase boundary, in a region where the effect of the sensor on the quantum many-body system is comparatively strong (blue dot), to illustrate the successful suppression of the effect upon introduction of a coupling cutoff.

One of the possible applications of Quantum Equilibrium Propagation (QEP) we proposed in the main text consists in phase recognition. In the experimental implementation, this would work by coupling a small quantum system (called the phase sensor) to a larger quantum many-body system, whose phases are to be recognized. During training, the internal couplings of the sensor as well as its couplings to the system will be updated. After training, the goal is to have a sensor that is able to indicate correctly the phase of the quantum many-body system, which can be changed when varying that system's parameters. Phase indication works by measuring some of the sensor degrees of freedom, such that the result represents a label for the phase.

During our initial numerical experiments, we observed that sometimes the system-sensor couplings become quite strong, comparable to couplings within the quantum many-body system itself. Although the performance of such a sensor can be nearly perfect, this has the side-effect of introducing relatively strong perturbations on the quantum many-body system. In this section, we analyze these perturbations and how they are suppressed when the coupling strength is reduced.

In Fig. S1, we show the spatial profiles of the spin operators inside the cluster Ising chain. In the absence of a sensor, these would all be spatially constant. The coupling to the sensor introduces perturbations in its vicinity. We observe that these deviations are strongest when the quantum many-body system is placed near a phase boundary, as might be expected from the usual behaviour of linear response near such boundaries.

We can suppress these effects, making them essentially insignificant, by introducing a cutoff on the system-sensor couplings during QEP training. If the cutoff is chosen sufficiently small (smaller than the typical couplings inside the system), we find that the effects become greatly reduced. In practice, we found best convergence and best results when the cutoff was implemented in a "soft" fashion. This was achieved by adding a term to the cost function that is of the type $\sum_j \lambda_{\text{cutoff}} \max(0, |\theta_j| - c)$. Here $|\theta_j|$ is one of the system-sensor couplings (we are summing over all of them), c is the value of the cutoff, and λ_{cutoff} represents the weight (i.e. the slope of the cost function term rising linearly as a function of coupling). For implementing a soft cutoff, we took $c = 0.1$ and $\lambda_{\max} = 0.02$, because this led to good convergence and small values of the resulting system-sensor couplings

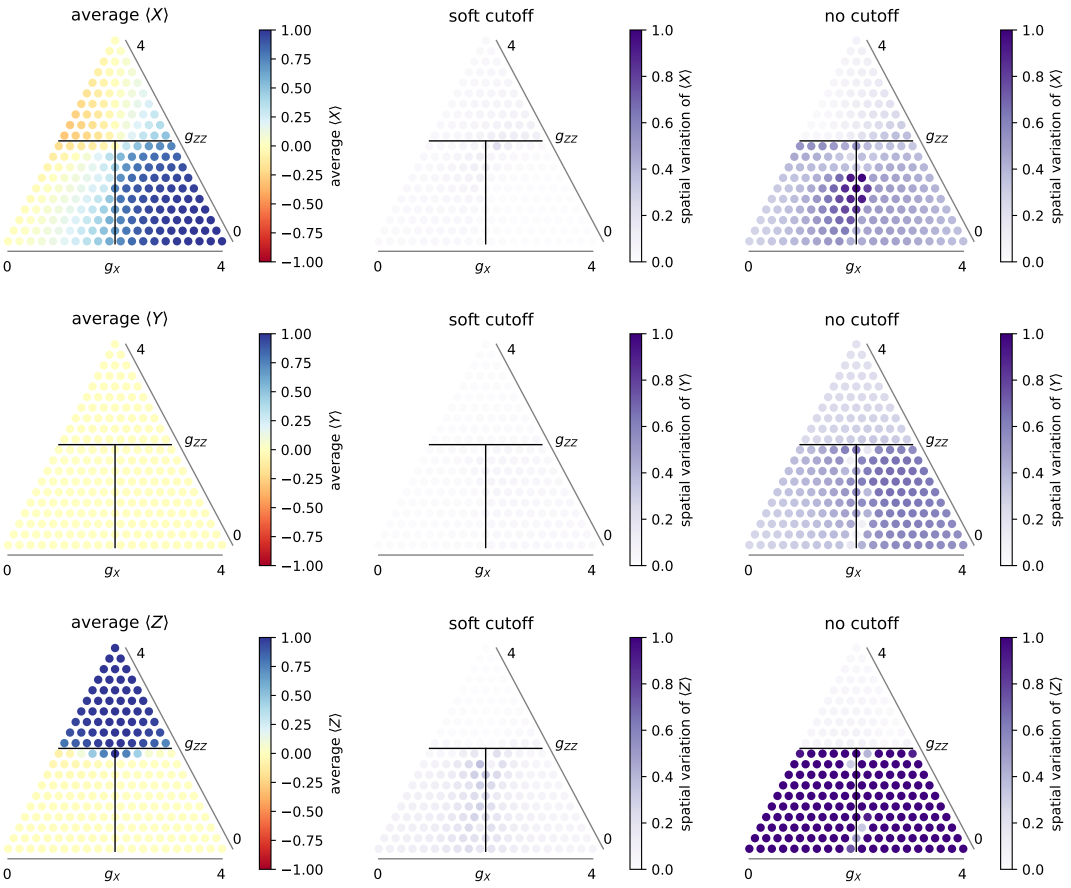

Supplementary Fig. S2. Phase recognition: Sensor effects across the phase diagram. We extract the 'spatial variation' in the cluster Ising chain, defined as the difference of maximum and minimum expectation value of a given operator, such as $\max_j\langle\hat{Z}_j\rangle - \min_j\langle\hat{Z}_j\rangle$. In the absence of a sensor coupled to the system, this would be zero, since the chain then is translationally invariant. We evaluate and plot this variation across the whole phase diagram. When the coupling is left unconstrained during QEP training, the variation is very large, especially in the Z spin component (which has a ferromagnetic coupling and can display strong fluctuations). This indicates that there are strong perturbations in the vicinity of the sensor. Imposing a cutoff on the system-sensor coupling reduces the variation to nearly zero everywhere. Leftmost panels: Spatially averaged expectation value across the phase diagram, for reference.

with correspondingly very weak effects of the sensor on the system behaviour, as demonstrated by the figures in this section. This soft cutoff was applied in all our numerical experiments reported in the main text (except for the analysis of gradients vs nudging and shot noise, which does not depend on these details since it is anyway performed in the early stages of training).

Furthermore, we have analyzed how these perturbations of the system due to the sensor depend on the location in the phase diagram. The results are shown in Fig. S2. We see that without cutoff, the perturbations can be large across whole ranges of the diagram.

III. FURTHER DETAILS ABOUT THE KITAEV HONEYCOMB MODEL

A. Implementation of the model

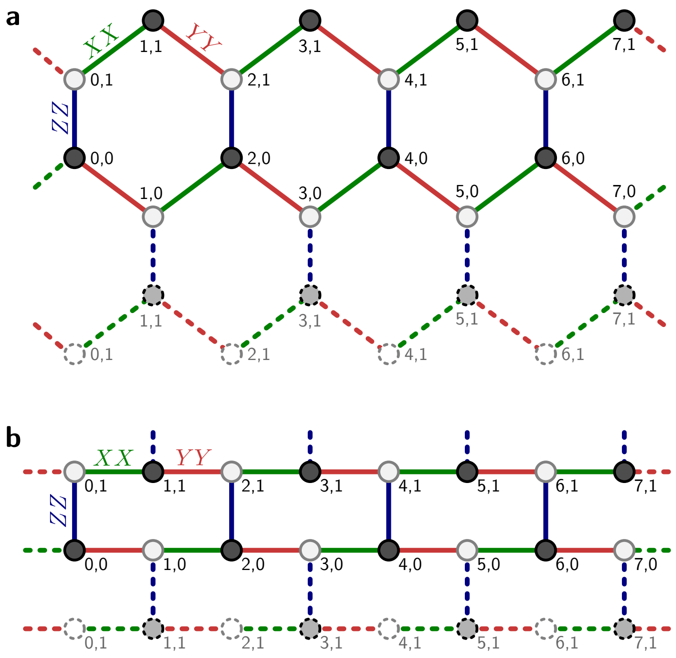

Supplementary Fig. S3. **Kitaev honeycomb model with the periodic boundary conditions we implemented.** **a** Hexagonal lattice and **b** brickwall representation of the lattice which we used to implement the model.

Fig. S3 **a** and **b** show the system we simulated numerically, including the boundary conditions we employed. The hexagonal lattice can be mapped to a brickwall lattice with the connectivity shown in Fig. S3 **b**.

B. Phase sensor

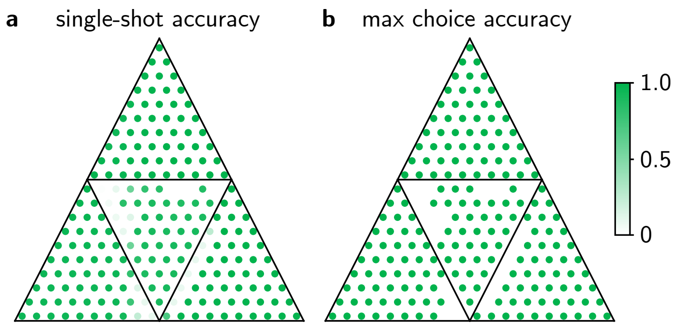

Supplementary Fig. S4. **a Single-shot accuracy and b max-choice accuracy in the phase diagram of the honeycomb Kitaev model.**

The training of the phase sensor consists of two steps: a pre-training phase and a second fine-tuning training phase which is shown in Fig. 3 of the main text. During the pre-training phase we train using the adam optimizer for 25 epochs with a learning rate of 0.01, for 5 epochs with a learning rate of 0.1, for 265 epochs with a learning rate of 0.05 and finally for 173 epochs with a learning rate of 0.01. To suppress the fluctuations in the cost functions in the second fine-tuning training stage, we use the adamax optimizer while keeping the learning rate at 0.01. During both training stages we assume small shot noise with $M = 10,000$. The nudging parameter was set to 0.05. We take the different sizes of the Abelian and non-Abelian phases into account such that during one epoch we sample the same number of points within each phase for one batch. In both training

phases, the batch size was set to 6, i.e., 3 points were sampled from each phase, respectively. We show the single-shot accuracy and many-queries ("max choice") accuracy across the phase diagram in Fig. S4 for the best-performing phase sensor. As we can see, the phase sensor for most parts can distinguish the Abelian and non-Abelian phase with a probability close to one and only fails close to the phase boundary.

IV. COMPARISON TO OTHER QUANTUM MACHINE LEARNING APPROACHES

So far, physics-based training of neuromorphic quantum systems has only been studied in the realm of so-called quantum machine learning. More specifically, we are talking of variational quantum circuits, which consist of a sequence of gate operations with continuous parameters, e.g. continuous rotation angles. While originally used for variational ground state search, they have also been employed to train quantum neural networks. However, we emphasize that this 'digital' quantum machine learning relies on a fully-fledged quantum computer, with fully controllable qubits (and recent indications are that it even needs to be fully fault-tolerant to achieve some quantum advantage, further increasing the requirements). The training technique there is the parameter shift method, with the slight improvement that parameters are not shifted infinitesimally but rather by $\pi/2$, creating better signal to noise ratio in the required measurements. Research of recent years has also indicated a problem with so-called barren plateaus, where the cost function is effectively flat in wide regions of parameter space, though this can be avoided to some extent by various ideas like cleverly constructed local cost functions.

In contrast to quantum machine learning (variational quantum circuits), quantum equilibrium propagation does not require a working quantum computer, only some experimental platform with many tuneable parameters, like an analogue quantum simulator. This drastically reduces the resource requirements and makes QEP training of large-scale experimental quantum many body systems much more likely in the short term. In addition, the QEP physical training method needs only a number of experiments of order 1 to extract the gradient with respect to all N trainable parameters, very much in contrast to variational quantum circuits, for which the number of required experiments scales like N.

Some other, less significant aspects are comparable between the two methods; e.g., the unavoidable projection shot noise implies that in both techniques a given experiment (for a given set of parameter values) needs to be repeated multiple times when a better estimate of the expectation values is desired.
